# Ornamental Phytoremediation in Cities: Context-Dependent Roles in Managing Potentially Toxic Elements

**DOI:** 10.3390/plants15040662

**Published:** 2026-02-22

**Authors:** Katalin Horotán, László Orlóci, Jana Táborská, István Dániel Mosonyi, András Neményi, Gábor Boronkay, Zsanett Istvánfi, Szilvia Kisvarga

**Affiliations:** 1 Institute of Biology, Eszterházy Károly Catholic University, 3300 Eger, Hungary; horotan.katalin@uni-eszterhazy.hu (K.H.); jana.taborska@uni-eszterhazy.hu (J.T.); 2 Ornamental Plant and Green System Management Research Group, Institute of Landscape Architecture, Urban Planning and Garden Art, Hungarian University of Agriculture and Life Sciences (MATE), 1223 Budapest, Hungary; nemenyi.andras.bela@uni-mate.hu (A.N.); boronkay.gabor@uni-mate.hu (G.B.); istvanfi.zsanett@uni-mate.hu (Z.I.); kisvarga.szilvia@uni-mate.hu (S.K.); 3 Doctoral School of Plant Sciences, Hungarian University of Agriculture and Life Sciences (MATE), 1223 Budapest, Hungary; 4 Department of Floriculture and Dendrology, Institute of Landscape Architecture, Urban Planning and Garden Art, Hungarian University of Agriculture and Life Sciences (MATE), 1223 Budapest, Hungary; mosonyi.istvan.daniel@uni-mate.hu

**Keywords:** urban PTEs, urban phytoremediation, ornamental plants, accumulation, stabilisation, translocation, green infrastructure

## Abstract

Potentially toxic element (PTE) contamination of urban soils poses long-term ecological and public health risks. Ornamental vegetation is increasingly discussed within green-infrastructure-based risk management. We screened and synthesised 167 field studies (>120 ornamental and horticultural plant species) to characterise the scope, reporting structure and design features of the available phytoremediation-related evidence. Studies assessed a mean of 3.21 elements (SD = 1.37); Pb, Cd and Zn were most frequently investigated (67%), whereas Ni, Cr and B occurred in <10%. Reported element richness differed by setting, averaging 3.8 ± 1.5 in wastewater-affected sites versus 2.6 ± 1.1 in urban parks. Using a study-by-element presence/absence matrix, co-reporting patterns separated three recurrent co-reporting profiles. The first three PCs explained 64.5% of variance (PC1: Pb–Zn–B; PC2: Cu–Ni; PC3: Cd–Cr). Accumulation was reported most often (56.8%), while stabilisation (17.9%) and translocation (25.3%) were less commonly addressed. For public space applications, accumulation-focused plantings require a defined maintenance pathway (pruning/harvest, biomass removal, and safe handling or disposal) to avoid recirculation of metal-bearing material within the urban environment. Sampling focused on aboveground tissues (73.4%) more than roots (28.9%). In multiple regression, environmental type was associated with element richness (Adj. R^2^ = 0.08, *p* = 0.001). Here, richness is treated as an index of reporting breadth. Overall, the dominant quantitative signals reflect context-dependent reporting and study design patterns. They do not represent harmonised, concentration-based remediation outcomes. These patterns provide an evidence map to support context-aware interpretation and future study standardisation.

## 1. Introduction

United Nations projections suggest that by 2050 nearly 70% of the global population will live in urban areas [1,2].

The expansion of transport networks, industrial activities and impervious surfaces has contributed to the build-up of pollutants in cities. Among these, potentially toxic elements (PTEs) such as Pb, Cd, Zn and Cu are important because they persist in soils and influence both ecosystem processes and human exposure [3,4,5]. Their strong affinity for soil colloids and the limited natural removal processes often result in long residence times. Industrial point-source contamination is often associated with legacy inputs and may extend into deeper soil horizons through burial [6], mixing, or site reworking, particularly in industrial and post-industrial areas [7]. By contrast, diffuse urban contamination is typically concentrated in upper soil layers and is sustained by traffic-related deposition (road dust, brake and tyre wear) and the wash-off of built surfaces [8]. Ornamental plantings interact primarily with this near surface and diffuse contamination domain via topsoil contact, rhizosphere processes and interception of particulate deposition [9,10].

Urban PTE distributions are highly heterogeneous in space, influenced by microtopography, land-use history and local management [11]. This variability complicates risk assessment and the design of mitigation strategies.

Green infrastructure has a dual role in the urban contamination context. It can reduce heat stress, improve air quality and provide social and aesthetic benefits, but it also acts as a sink for PTEs [12,13]. Understanding contaminant fluxes therefore requires attention to the plant–soil–atmosphere system.

Phytoremediation—the use of plants to extract, stabilise or transform contaminants—is often discussed as a complementary, site-specific option for urban soil management [14,15]. Evidence from urban parks, roadside strips and wastewater-affected green zones has increased in recent years, but coverage remains uneven across site types and regions [16,17].

Ornamental plants may bridge remediation aims and landscape functions. Many species combine abiotic stress tolerance with a measurable capacity to accumulate or immobilise PTEs, and they are already widely used in managed green spaces [18,19,20]. For example, *Tagetes erecta* has been reported to accumulate Cd, while *Pelargonium hortorum* may contribute to Pb immobilisation in the rhizosphere [21,22].

Evaluating ornamental species for urban phytoremediation requires an integrated perspective drawing on soil chemistry, plant physiology and urban ecology. Indices such as the bioconcentration factor (BCF) and translocation factor (TF) allow comparison among species and help distinguish plants more suited to phytostabilisation from those with higher phytoextraction potential under given conditions [23,24]. Simple quantitative summaries of these indicators can also highlight where evidence is strong, where it is biassed towards specific tissues or mechanisms, and which urban environments remain under-represented [25].

Phytoremediation in urban environments has attracted increasing attention as a potentially low-cost, nature-based solution that can be integrated into green infrastructure. However, evidence is dispersed across heterogeneous field contexts and often mixed with controlled experiments, limiting direct transfer to urban planting practice. The primary aim of this review is to synthesise field-based evidence on the phytoremediation potential of ornamental and horticultural plant species in urban environments, with specific focus on how environmental context, element mixtures and methodological choices shape reported outcomes. Specifically, we aim to evaluate how reported outcomes for accumulation, stabilisation and translocation differ across environment types (parks, roadside belts, wastewater-affected and industrial/post-industrial sites) and PTE mixtures, and to use this evidence to support context-adapted selection of ornamental species for urban green infrastructure plantings. This review deliberately restricts the evidence base to field studies conducted in explicitly urban settings and focuses on ornamental/horticultural taxa, thereby complementing broader phytoremediation overviews dominated by controlled experiments and non-ornamental species. Across 167 urban field studies, we quantify how reported outcomes vary with environmental context, PTE mixtures and study design choices. We further propose an evidence-based typology of urban PTE reporting profiles to support context-adapted planting strategies, rather than generic species rankings.

### 1.1. Potentially Toxic Element Contamination in Urban Environments

Urban environments show complex contamination patterns linked to transport, industry, construction and waste management [26,27]. Compared with agricultural or natural soils, urban soils are often disturbed and stratigraphically heterogeneous, with anthropogenic materials and strong small-scale variability. The persistence of PTEs, together with high variability in their mobility, remains a central challenge for urban soil research and risk-based management [5,28].

#### 1.1.1. Sources of PTEs in Urban Ecosystems

Urban PTE loads arise mainly from human activities whose intensity and spatial concentration increase with urban density, affecting soils, water and air through direct and diffuse emissions [29]. Several global assessments show that PTE concentrations in urban soils often exceed rural background levels, with clear implications for large urban populations [30]. One synthesis suggested that Pb and Cd can exceed health-based guideline values in many cities [31]. Traffic remains one of the most important sources, with emission intensity and accumulation patterns scaling with traffic density and the surrounding built environment. Brake and tyre wear emit Cu-, Zn- and Pb-bearing particles, and fuel combustion and vehicle components add Pb, Ni, Cd and Cr [32,33].

Industrial activities, including metal processing and construction material production represent density-dependent point and area sources of Pb, Zn and Cr, whose influence often extends beyond immediate source areas [34]. Construction and demolition waste add diffuse inputs through weathering and leaching of paints, concrete dust and metallic components [35]. Waste management practices, such as composting of contaminated materials and irrigation with wastewater, can further increase PTE levels, especially where urban agriculture is expanding [36,37].

In many regions, particularly outside highly urbanised economies, rural settlements and small towns still dominate over large metropolitan areas [38]. In such contexts, major sources of potentially toxic elements are often linked to fossil fuel extraction, mining activities and other pollution-intensive industries [39], including oil and gas production, coal mining and associated processing facilities [40]. Unlike traffic-dominated urban settings, these activities generate contamination that is spatially extensive rather than density-driven, affecting soils through atmospheric deposition, wastewater discharge and accumulation of extraction- and processing-related residues [41]. As a result, substantial PTE loads can occur even in areas with relatively low population density, often in close proximity to agricultural land and small settlements [42,43].

Climate extremes may alter these pathways by affecting mobilisation and re-suspension of previously deposited elements [44,45]. Urban density provides a more informative organising framework than a simple urban–non-urban distinction, because it integrates traffic intensity, built-up surface ratio and the spatial concentration of anthropogenic activities. Density-dependent gradients therefore offer a mechanistic basis for interpreting variability in potentially toxic element accumulation across different urban environments.

The mobility and bioavailability of PTEs depend strongly on soil properties such as pH, redox conditions, texture, and organic matter content [46]. In acidic soils, Cd and Zn usually show higher solubility, whereas higher organic matter can favour immobilisation of Pb and Cu [47,48].

In urban soils, PTEs are often concentrated in the upper 10–20 cm due to atmospheric deposition and surface runoff [49]. This depth corresponds to the main rooting zone, where phytostabilisation or uptake can influence exposure pathways. Reported concentrations in parks, playgrounds and gardens vary widely among cities, reflecting differences in land-use history and emission sources [50,51,52].

Disturbances such as excavation, construction and extreme rainfall can redistribute PTEs vertically and further complicate risk assessment [53,54]. These patterns support risk-reduction strategies that combine vegetation with soil amendments where needed [55,56].

#### 1.1.2. Environmental and Human Health Risks

PTE contamination can alter soil microbial communities, reduce enzyme activity and disrupt key biogeochemical cycles, lowering the functional capacity of urban soils [57,58].

Human exposure occurs through inhalation of contaminated dust, incidental ingestion of soil and consumption of plants grown in contaminated substrates [59]. Lead is a major concern due to its neurotoxicity, particularly for children [60,61]. Cadmium exposure is cumulative and contributes to chronic health problems; interactions among elements are not fully captured in many risk models [62,63].

**Figure 1:**
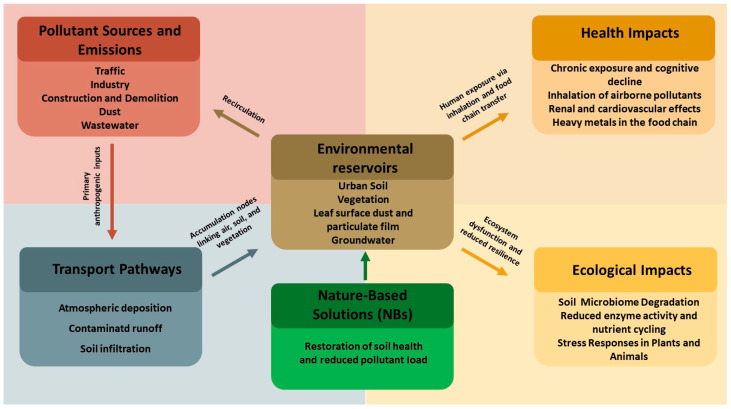
Conceptual framework of urban PTE pollution and nature-based solutions. Schematic showing how major urban PTE sources and emissions feed transport pathways and environmental reservoirs. These reservoirs mediate human and ecological impacts. Nature-based solutions, including vegetation-based phytoremediation, can reduce PTE loads in these reservoirs and thus lower both health and ecological risks.

These considerations support integrated assessment approaches that consider total concentrations, bioavailability, exposure scenarios and land use to guide management [64,65], and they motivate nature-based solutions that address the whole source–pathway–impact chain (Figure 1).

#### 1.1.3. Urban Soil Properties: pH and Physical Constraints

Urban soils differ from agricultural and natural soils not only in contamination patterns but also in their physical and chemical properties [66,67]. A common feature of many urban soils is elevated pH, often exceeding 7.5, driven by inputs of cement and concrete dust, building debris and alkaline construction materials [68,69]. In addition, urban soils are frequently compacted, structurally disturbed and heterogeneous, which constrains root growth, limits water infiltration and alters redox and microbial processes relevant to metal mobility [67].

Soil pH strongly controls the solubility and bioavailability of many potentially toxic elements [70]. At alkaline pH, metals such as Zn and Cu tend to precipitate or become strongly sorbed to mineral phases, reducing their mobility and uptake by plants [71]. Under such conditions, phytoextraction-oriented approaches are inherently limited, regardless of plant species, because metal availability rather than plant capacity becomes the dominant constraint [72]. In contrast, phytostabilisation strategies that rely on immobilisation and containment in the root zone may remain effective under high-pH urban soils.

These properties have direct implications for species selection and interpretation of reported performance. Many ornamental species used in cities are selected for tolerance to compacted, alkaline substrates rather than for optimal nutrient acquisition [73]. Reported accumulation under field conditions should therefore be interpreted in the context of local soil chemistry, particularly pH, and not extrapolated to settings where metal availability differs [74]. Explicit consideration of urban soil pH and physical constraints is essential for distinguishing between contexts where phytoremediation outcomes are plant-limited and those where they are soil-limited [75].

### 1.2. Phytoremediation Potential of Ornamental Plants in Urban Environments

#### 1.2.1. Phytoremediation-Relevant Functions and Exposure Considerations

Urban vegetation can reduce pollution through capture of airborne particles, microclimate effects and changes in soil processes. Leaf traits such as wax layers, surface roughness and trichomes enhance capture of particle-bound elements and can reduce resuspension [76].

Trees, shrubs and herbaceous covers play different roles. Species such as *Betula pendula*, *Acer platanoides*, *Salix integra* and *Populus nigra* have been reported to accumulate or immobilise Pb, Cd and Zn in leaves, bark or rhizosphere compartments [55,77]. Fast-growing *Populus* and *Salix* taxa often show higher translocation capacity, which may support phytoextraction in suitable settings [78]. In contrast, *Quercus robur* and *Tilia cordata* tend to retain more metals in roots, which fits phytostabilisation strategies [79].

Shrubs are widely used in roadside belts where they help trap dust and stabilise surface soils [80,81]. Evergreen ornamentals such as Buxus sempervirens and *Mahonia aquifolium* have been reported to accumulate Pb and Zn in traffic-impacted sites [82]. *Forsythia* × *intermedia* and *Ligustrum vulgare* have been described as tolerant accumulators of Cd and Cu in urban soils [83,84].

Herbaceous ground covers and grasses form dense root mats that reduce erosion and can limit movement of surface-deposited elements. *Festuca arundinacea*, *Lolium perenne* and *Trifolium repens* are often mentioned for their tolerance and stabilisation roles [85,86,87,88].

#### 1.2.2. Phytoremediation Potential of Ornamental Plants

Urban vegetation is shaped by management-driven species selection, which affects the functional diversity and ecosystem services of green spaces [89,90]. Ornamental plants are chosen for appearance, adaptability and stress tolerance, but these traits can also support use in contaminated substrates [20,91]. Unlike many classical hyperaccumulator species, which often have low biomass and limited landscape value, ornamentals can offer a practical balance between visual quality and remediation potential [92].

**Figure 2:**
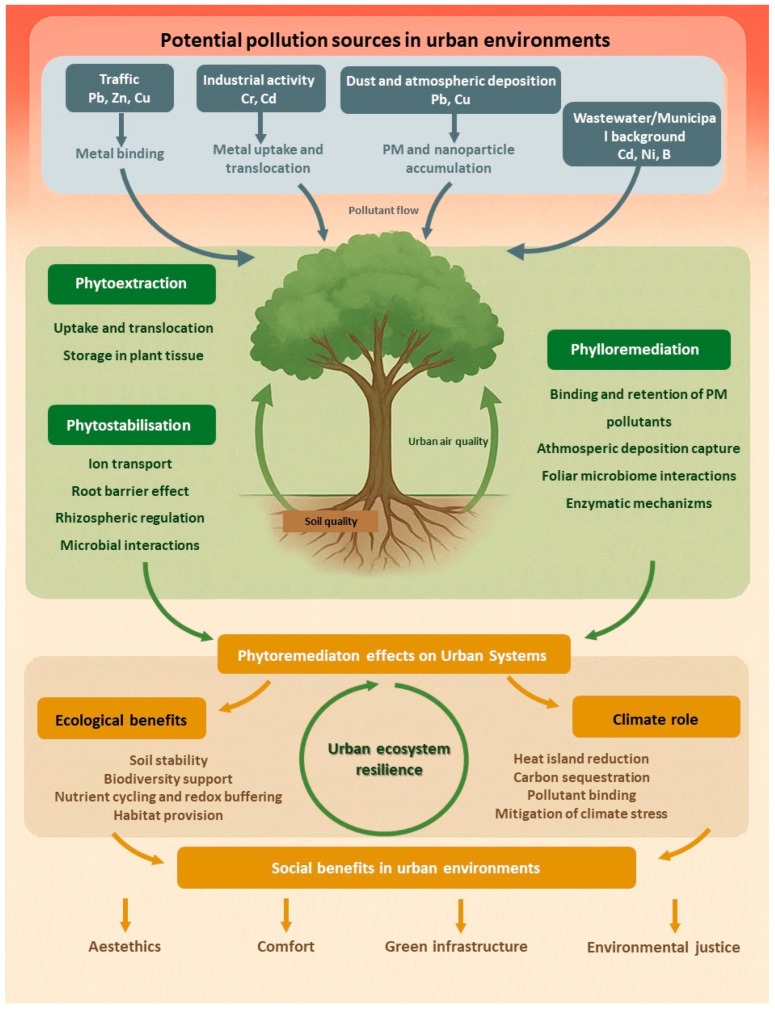
Urban phytoremediation as an integrated nature-based system. Conceptual diagram illustrating how ornamental vegetation in cities mediates pollutant fluxes and ecosystem responses.

Urban contamination often co-occurs with heat, drought, salinity and soil compaction, so ornamental performance depends on combined morphological, physiological and biochemical strategies (Table 1). These strategies are linked to wider phytoremediation and ecosystem service functions (Figure 2).

Compared with the wider phytoremediation literature, which is still dominated by pot, hydroponic and laboratory trials and by non-ornamental taxa [5,91], this review focuses only on field-based studies on ornamental and horticultural species in clearly urban settings. This scope allows us to track how research priorities are shifting from hyperaccumulator-centred experiments towards plantings that are embedded in real green infrastructure. By combining publication year, plant functional type and mechanism categories, our analysis adds a practice-oriented perspective to earlier, more bibliometric overviews.

## 2. Results

**Figure 3:**
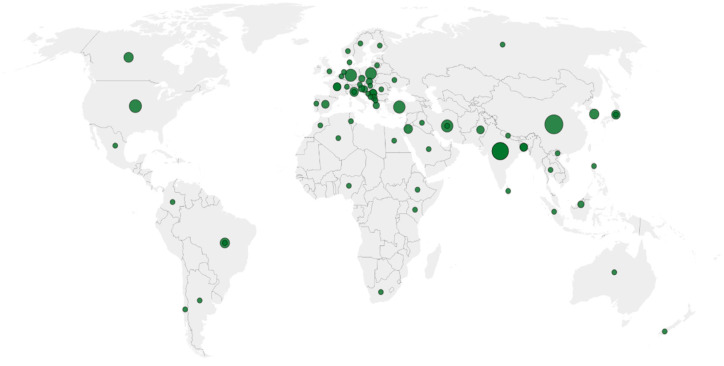
Geographical distribution of urban phytoremediation field studies included in the synthesis. Bubble map showing the locations of the 167 studies analysed, with bubble size proportional to the number of studies per city or region. Research activity is concentrated in parts of Asia and Europe, with relatively fewer field-based studies reported from North America, Africa and Oceania.

After systematic screening, 167 peer-reviewed field studies on phytoremediation-related processes in urban ornamental and horticultural plants were included. Most studies were carried out in urban parks, roadside green belts and wastewater-affected areas. These settings reflect the main situations where ornamental vegetation is routinely used and exposed to multi-source urban contamination (Figure 3).

Across the literature, the most frequently assessed PTEs were Pb, Cd and Zn. On average, studies evaluated 3.21 elements (SD = 1.37), indicating that urban field research usually addresses multiple-element settings rather than single-element exposure. Less common elements (e.g., Ni, Cr, B) appeared in a smaller subset of studies, so the current evidence base is still centred on a core set of urban PTEs.

Mechanism focus was uneven. Accumulation was most frequently investigated, while stabilisation and translocation were addressed less often. This pattern follows the dominant use of aboveground tissue sampling and shows that root-zone and rhizosphere processes are less represented in field work. Together, these findings suggest that our current knowledge on ornamental phytoremediation in cities is shaped both by site conditions and by measurement choices that favour aboveground outcomes.

Regression analyses suggest that reported PTE richness varied with site context, reflecting differences in which elements are assessed and reported across urban settings. Publication year alone was not associated with the number of elements assessed (F(1,167) = 0.009, *p* = 0.924). In contrast, the multiple regression model including year, plant type, environment and mechanism focus was significant (F(4,164) = 4.651, *p* = 0.001) but explained only a small proportion of variance (Adj. R^2^ = 0.08) and is therefore interpreted as exploratory with respect to reporting breadth. Environment showed the largest standardised coefficient (β = −0.229, *p* = 0.004), suggesting that variation in reported PTE richness is more closely associated with environment category than with plant growth form or mechanism focus within this coarse categorical framework. Differences among environments are summarised in Table 2, which indicates higher median reported richness in wastewater-affected and industrial sites than in parks and roadside belts, consistent with the direction of the regression results.

## 3. Discussion

### 3.1. Trends and Shifting Priorities in Urban Phytoremediation

In recent years, there has been an increase in field studies on phytoremediation-related processes in urban ornamental and horticultural plants. In our dataset, many studies were published after 2015, with peaks in 2023–2024, which likely reflects growing interest in urban soil quality and the role of vegetation in exposure management. However, the distribution of study sites shows important gaps. Roadside green belts, wastewater-affected areas and parks dominate the literature [102,103], whereas playgrounds, residential lawns and community gardens are less often studied, even though these are places where people, especially children, have frequent contact with soil. This limits how far current results can be applied to the most sensitive urban spaces.

A similar imbalance appears in mechanism focus. Accumulation is reported most often, while stabilisation and translocation receive less attention. The strong preference for aboveground sampling may overemphasise shoot-based outcomes compared with rhizosphere processes. The dominance of Pb, Cd and Zn in urban ornamental studies is consistent with expected PTE profiles, but it also suggests that multi-element interactions and less frequently reported PTEs (e.g., Ni, Cr) remain underexplored. Taken together, the concentration of ornamental field studies in the last decade and the known dominance of controlled experiments and non-ornamental taxa in older phytoremediation literature [5,91] support the idea that research is gradually shifting from hyperaccumulator-focused lab work towards real-world, urban applications.

### 3.2. Functional Plant Types and Strategic Differences in Urban Phytoremediation

The performance of ornamental species can be understood in terms of functional plant types defined by growth form, lifespan and root architecture [92]. In our synthesis, herbaceous seasonal plants were most frequently studied, whereas woody ornamentals and other long-lived types were less common. This likely reflects practical research constraints but may also bias the evidence away from taxa that are most relevant for long-term PTE management.

Perennial grasses and legumes deserve special attention. *Festuca* and *Lolium* species are often linked to stabilisation potential through dense root systems and rapid ground cover [104]. *Trifolium repens* may support rhizosphere processes that complement PTE immobilisation [105,106]; yet these groups appear less often than fast-growing annual ornamentals, even though they may be more useful in low-to-moderate contamination where exposure reduction is the main goal. Woody ornamentals, such as Salicaceae and Oleaceae taxa, combine higher biomass and deeper rooting and may contribute to both stabilisation and, in some cases, translocation-oriented strategies [106]. Their reported performance matches the dominant Pb–Cd–Zn profiles in our dataset [107,108]. However, uneven sampling of root-zone processes across growth forms limits robust comparisons among these types.

**Figure 4:**
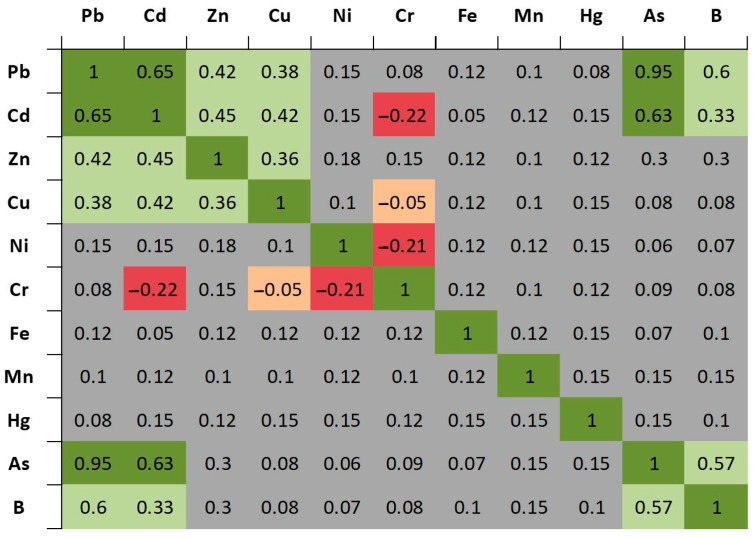
Pearson correlation matrix of PTEs reported in urban phytoremediation studies. Heatmap of Pearson correlation coefficients among commonly assessed potentially toxic elements (Pb, Cd, Zn, Cu, Ni, Cr, Fe, Mn, Hg, As, B) across 167 field studies. Green cells indicate positive correlations and red cells negative correlations, with more saturated colours denoting stronger relationships; grey cells denote weak or no correlation. The matrix highlights, for example, strong positive associations between Pb and As or B, and negative associations between Cr and Zn, consistent with differences in co-reporting patterns in different urban settings. Correlation coefficients quantify co-reporting patterns across studies rather than concentration-based co-variation. Correlations were calculated using Pearson’s product–moment correlation coefficient based on presence–absence and co-reporting frequencies of PTEs across the 167 included field studies.

Taken together, the co-reporting structure of PTEs across the included studies highlights heterogeneous element mixtures, which supports the use of functionally diverse plantings when the management goal is exposure reduction under mixed urban contamination profiles (Figure 4).

### 3.3. Regional Differences and Patterns of Urban PTE Pollution

Our synthesis confirms that Pb, Cd and Zn are the most frequently reported PTEs in field studies of urban ornamental vegetation. This aligns with frequent relevance of traffic, industrial legacies and ageing infrastructure in many cities [109,110]. Differences in element combinations across environments suggest that urban PTE profiles are strongly context-dependent and shaped by land-use history and source mixtures.

**Figure 5:**
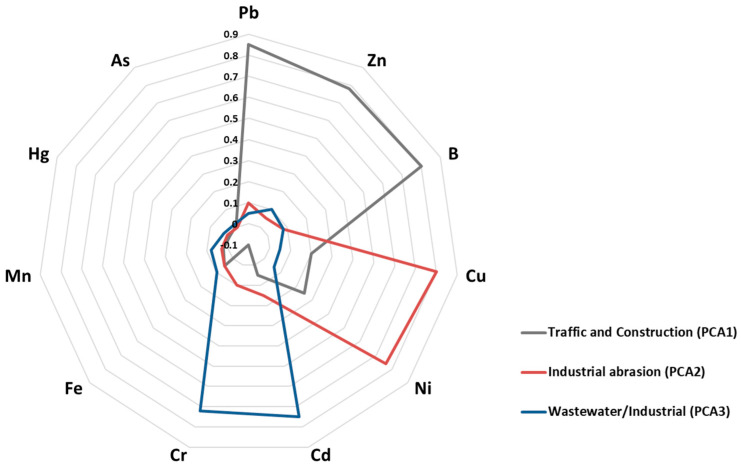
Principal component analysis (PCA) loadings for PTEs across urban field studies based on a study-by-element presence/absence matrix (Pb, Cd, Zn, Cu, Ni, Cr, Fe, Mn, Hg, As, B). Radar plot summarising loadings of individual elements on the first three principal components. Components are labelled by their dominant loadings (PC1: Pb–Zn–B; PC2: Cu–Ni; PC3: Cd–Cr). Because the input indicates whether elements were analysed (not harmonised concentrations), the ordination is interpreted as co-reporting structure in the evidence base. It is not interpreted as source apportionment, mechanistic coupling, or comparative uptake performance.

Correlation and PCA analyses show recurring combinations of PTEs. Because the analyses rely on presence/absence coding, the resulting patterns should be seen as signals of co-reporting rather than direct evidence of shared uptake mechanisms (Figure 5). The same results should also not be read as evidence of shared sources or “mixtures.” In this framework, Pb–Zn–B, Cu–Ni and Cd–Cr are recurrent co-reporting profiles. They describe how studies tend to group elements across research contexts. Any links to traffic-, industrial- or wastewater-related settings should be interpreted as contextual study-focus patterns, not as source apportionment. These patterns agree with previous work showing that different urban sources can create recognisable PTE signatures at site level [111]. However, our synthesis does not quantify sources or contamination magnitude.

From a planning perspective, these findings are consistent with the value of context-adapted strategies. Species selection and mechanism priorities should respond to the main local PTE mixtures and to the management goal—whether it be long-term stabilisation and exposure reduction, or, where feasible, targeted biomass removal under moderate contamination.

Our PCA-based grouping adds to earlier source-apportionment studies by deliberately ignoring concentration magnitudes and focusing instead on recurring combinations of elements recorded in field-based ornamental studies. In practice, these axes can serve as a simple descriptive guide: sites with Pb–Zn–B signals (PC1) are often reported in traffic- and construction-related mixtures where species with strong dust-capture and surface-stabilisation traits are relevant, whereas Cd–Cr-dominated profiles (PC3) are more frequently reported in wastewater-affected or industrial contexts where belowground tolerance and stabilisation traits may be prioritised.

### 3.4. Methodological Biases and Complementary Mechanisms in Urban Phytoremediation

**Figure 6:**
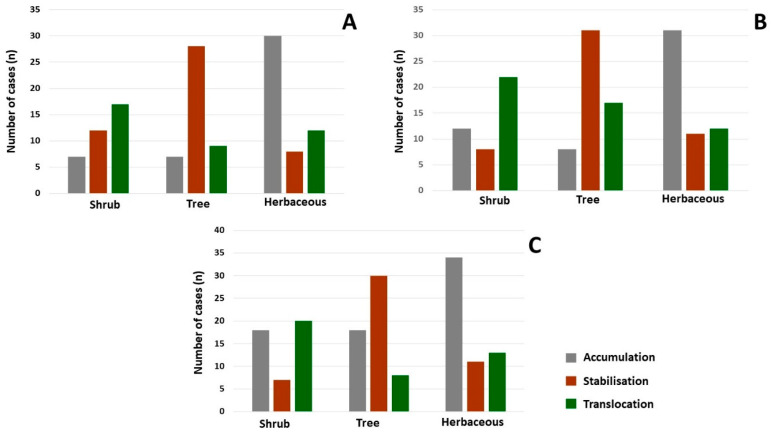
Distribution of phytoremediation mechanisms across plant types in different urban environments. Number of reported cases (n) for accumulation, stabilisation and translocation for shrubs, trees and herbaceous species in (**A**) parks, (**B**) roadside green belts and (**C**) wastewater-affected areas. The panels illustrate the overall dominance of accumulation-focused studies and the comparatively low representation of stabilisation and translocation, particularly for root-zone processes and woody ornamentals.

Among the 167 studies, 56.8% identified accumulation as the main phytoremediation mechanism, while stabilisation and translocation accounted for 17.9% and 25.3% of cases, respectively. The distribution of mechanisms across growth forms and environment types is illustrated in Figure 6. Measuring PTE concentrations in aboveground tissues is usually easier than assessing rhizosphere stabilisation, which likely contributes to the dominance of accumulation-focused evidence [22,91,107].

Our dataset confirms this imbalance: 73.4% of studies assessed shoots and leaves, while root-zone profiles were included in only 28.9%. Similar patterns have been observed elsewhere, where aerial tissues are overrepresented relative to rhizosphere processes [109,112,113]. As a result, current field-based literature may understate longer-term stabilisation functions that are important for exposure reduction [48,114].

Accumulation was especially common in herbaceous species, but woody ornamentals also show important uptake [109,115]. *Salix integra* and *Populus nigra* were among the taxa with notable accumulation in the reviewed studies, indicating that PTE uptake is not limited to fast-growing annuals. In some cases, *Salix* species also showed TF > 1 for Pb, and Tagetes erecta recorded relatively high Cd concentrations in shoots [116]. These examples show that accumulation and translocation can co-occur in ornamentals, but their relative importance depends on site conditions and plant type.

Stabilisation is still less frequently described in detail. *Festuca arundinacea* and *Trifolium* repens were among the most often cited taxa in stabilisation-focused studies, consistent with their dense roots and rhizosphere activity [106,114]. Publication preferences may also favour accumulation results with clear aboveground signals.

Overall, accumulation, stabilisation and translocation should be seen as complementary processes. We suggest that future urban field studies, at a minimum, include paired shoot–root sampling and basic soil characterisation (pH, organic matter, texture and at least one indicator of bioavailable PTEs). This minimum dataset would improve comparability, help separate accumulation from stabilisation and provide a stronger basis for mechanism-oriented syntheses [15,22].

#### Rhizosphere Interactions and Microbial Mediation of Phytoremediation

Rhizosphere processes play a central role in determining plant performance and metal behaviour in contaminated urban soils, yet they remain underrepresented in field-based ornamental phytoremediation studies [117]. Mycorrhizal fungi and plant-growth-promoting rhizobacteria (PGPR) influence metal mobility, nutrient acquisition and stress tolerance through multiple pathways [118], including changes in rhizosphere pH, organic acid production, chelation, and modulation of plant antioxidant responses [119,120]. These interactions can enhance plant establishment and persistence under conditions where chemical toxicity, compaction and nutrient imbalance would otherwise limit growth [121].

In urban contexts, microbial mediation may be particularly important because soil conditions are often unfavourable for root development and metal uptake [122]. Evidence from field and semi-field studies indicates that mycorrhizal associations can increase plant tolerance to PTE exposure and, depending on the context, either promote metal immobilisation in the root zone or facilitate controlled uptake [123]. Similarly, PGPR can support root growth and reduce metal-induced stress, indirectly improving phytostabilisation performance [124] even where phytoextraction remains constrained by low metal availability at high pH [125].

Microbial inoculation has therefore been proposed as a complementary strategy to improve the functional performance of ornamental plantings on contaminated urban soils [126]. However, its effectiveness depends strongly on site conditions, host–microbe compatibility and long-term persistence under urban management regimes. In practice, microbial amendments should be viewed as context-dependent enhancers rather than universal solutions, and their application requires field validation under realistic urban conditions [127].

### 3.5. Site-Specific Pollution Pathways and the Role of Ornamental Plant Strategies

The type of urban environment appears to be a key correlate of reported PTE profiles and reporting breadth. In our dataset, wastewater-affected areas represented 28.4% of sites, roadside belts represented 34.3%, and urban parks represented 22.5%. Across these categories, studies differ in which elements they assess and report. Given our study-level presence/absence coding, we interpret this as evidence-structure variation rather than separation in contamination load [25,28,29].

Across site types, Pb, Cd and Zn were most frequently reported. Wastewater-affected settings were more frequently reported with combined Cd–Pb–Zn profiles, while roadside environments more often reflected traffic-related mixtures [39,64]. Urban parks generally showed lower PTE richness, though multi-element signatures were still reported in some cases. We interpret these differences as differences in study focus and reporting conventions across settings. They are not treated as direct evidence of source mixtures or contamination magnitude [50,128].

Consistent with this interpretation, PCA loadings highlight Cu, Zn, Pb and B among the elements contributing to separation in co-reporting space across environment categories [25,112].

From a practical standpoint, these results are consistent with site-adapted ornamental phytoremediation. Wastewater-impacted areas may prioritise species with higher tolerance and measurable accumulation under field conditions [15,107]. Roadside zones may benefit from taxa that combine surface cover with stabilisation traits. Parks can integrate long-lived woody ornamentals that align with stable, low-maintenance exposure management along with aesthetic functions [13,16,92].

### 3.6. PTE Richness as an Indicator of Urban Pollution Complexity

PTE richness—the number of distinct PTEs reported per study—offers a simple summary of contamination complexity in urban ornamental research. The reviewed studies reported an average of 3.21 elements (SD = 1.37), with values from 1 to 7. This range fits the heterogeneous nature of urban contamination, where traffic, industrial legacy, construction inputs and wastewater exposure can overlap [11,29]. Temporal analysis showed no significant link between publication year and the number of elements investigated (F(1,167) = 0.009, *p* = 0.924). This suggests that, despite increased research activity, the breadth of assessed PTEs still focuses mainly on a small set of high-priority elements, especially Pb, Cd and Zn [33,65,128].

The multiple regression including year, plant type, environment type and mechanism focus was significant (F(4,164) = 4.651, *p* = 0.001; Adj. R^2^ = 0.08). Environment type was again the largest association with PTE richness (β = −0.229, *p* = 0.004). Wastewater-affected and industrially influenced settings showed higher mean PTE richness than parks or roadside zones. Plant type and mechanism had weaker effects, likely because species and mechanism categories overlap across studies and our categories are coarse [65,128]. Although the explained variance is modest (Adj. R^2^ = 0.08), this magnitude is expected in heterogeneous, study-level syntheses where unreported site covariates (e.g., pH, organic matter, bioavailable fractions) add substantial noise [4,47,124]. The inference is descriptive and comparative; it is not causal.

These findings support the view that reported PTE richness in ornamental phytoremediation studies is shaped mainly by site context and reporting choices. Future field work would benefit from more consistent reporting of soil properties and root-zone endpoints to better understand the ecological drivers behind these patterns [22,48,114].

### 3.7. Limitations and Strengths

This review focuses on field-based studies of urban ornamental and horticultural plants to capture evidence generated under real site conditions. The dataset includes 167 studies and more than 120 species, providing a broad base to identify recurring PTE profiles, mechanism emphasis and context-related patterns.

Several limitations should be noted. First, methodological heterogeneity limits direct comparability. Differences in sampling strategies (shoot vs. root), analytical methods (AAS, ICP-OES, ICP-MS) and reported endpoints all add uncertainty [15,22]. Second, key contextual variables—such as soil pH, organic matter and bioavailable PTE fractions—were often missing, which restricted more detailed multivariate analysis. Third, the presence/absence coding used for some variables simplifies ecological gradients and does not capture dose–response relations or interactions among elements [22,112]. Finally, publication bias may favour accumulation-focused studies with clear aboveground signals, reinforcing the under-representation of stabilisation-oriented work [5,28].

The main strengths of this review are the field-based focus and the integration of several analytical views to describe evidence patterns across urban contexts [13,114]. The inclusion of under-represented functional groups, such as perennial grasses and shrubs, also supports a more practice-oriented view of ornamental systems in urban green infrastructure [108].

### 3.8. Future Research Directions and Practical Implications

Practical guidance for green infrastructure planning in PTE-affected urban sites should be framed as a decision process that links site context, exposure pathways and realistic management capacity, rather than as a generic species-ranking exercise. In most public space settings, the most defensible near-term objective is exposure mitigation through containment and stabilisation [129], because extraction-oriented designs only translate into risk reduction if biomass removal and safe handling are explicitly planned and implemented over multiple maintenance cycles [130]. Accordingly, plantings should be selected and arranged to minimise resuspension and direct contact with contaminated substrates [131,132], to reduce off-site transport by runoff, and to maintain stable ground cover under drought, heat and trampling pressures typical of urban use [133].

For practitioners, a minimum site screen is essential before design choices are made, because PTE behaviour and bioavailability are strongly modulated by local soil properties and land-use history [134]. At a minimum, projects should document the expected PTE mixture, soil pH, organic matter status, texture-related constraints and basic hydrologic behaviour [135], together with the dominant human contact routes at the site, including inhalation of resuspended dust and direct soil contact in high-use areas [136]. Where the intended users include children or where contact intensity is high, designs should prioritise physical separation from bare soil and durable, continuously vegetated surfaces, and they should avoid interventions that increase dust formation or mobilise fine particles during dry periods [137,138].

Planting composition should be treated as a functional portfolio that combines woody ornamentals, herbaceous species and perennial ground covers [117], because heterogeneous urban PTE profiles rarely align with single-mechanism designs. Aboveground sampling has dominated the literature, but rhizosphere and root-zone processes are central to stabilisation and longer-term containment [139], so field projects should incorporate root-zone profiling as part of monitoring whenever feasible. Finally, expectations should be explicitly time-scaled: plant-based interventions act on multi-year horizons, and applied claims require repeated measurements across seasons and maintenance events, with transparent reporting of soil properties and root–shoot sampling that allows cross-site comparison without implying guaranteed remediation outcomes [140,141].

Our findings indicate that effective ornamental phytoremediation in cities depends on matching plant selection and management to site-specific PTE mixtures and risk-management goals. In most urban green infrastructure settings, the practical objective is exposure reduction, so phytostabilisation and surface containment are typically more feasible than extraction-focused designs [15]. Where phytoextraction is pursued, it should be restricted to contexts with defined harvest and disposal pathways; otherwise, accumulation may transfer PTEs into biomass without net risk reduction [128]. The dominance of Pb, Cd and Zn across environments suggests that future applications should prioritise strategies designed for these common urban profiles, while also better covering less frequently assessed PTEs and mixed-element interactions [29].

Future field studies should place more emphasis on traits linked to belowground processes, including root architecture, rhizosphere chemistry and plant–microbe interactions. The limited representation of stabilisation endpoints shows the need for more systematic root-zone profiling and standardised reporting of soil properties that influence PTE mobility and bioavailability [142].

From an applied perspective, diverse plantings that combine woody ornamentals, herbaceous species and perennial ground covers are likely to match heterogeneous urban PTE patterns better than single-species designs [112]. Such assemblies can combine visual acceptability with complementary functions: surface cover, immobilisation potential and, where appropriate, measurable uptake into harvestable biomass.

Overall, our results support a shift from searching for “best” species towards designing functionally diverse, site-specific plant systems for defined urban environments. This approach is more likely to improve long-term exposure reduction and to integrate ornamental phytoremediation into practical urban green infrastructure planning.

### 3.9. Biomass Management and Maintenance Constraints in Urban Phytoremediation

Management of metal-bearing biomass represents a critical practical constraint for ornamental phytoremediation in urban environments and needs to be addressed explicitly whenever accumulation-based strategies are proposed [143,144]. In public green spaces, routine maintenance activities generate recurring biomass streams through leaf fall [145], mowing residues, pruning cuttings and the removal of senescent annuals [146]. In accumulation-focused plantings, these materials often contain elevated concentrations of potentially toxic elements, particularly when uptake is reported primarily in aboveground tissues [147,148,149]. If such biomass is left on site or enters standard green-waste handling, metals may be redistributed locally through litter decomposition, incorporation into topsoil and the mobilisation of fine organic particles and dust, thereby undermining exposure-mitigation objectives [149,150].

Composting does not eliminate metals and therefore cannot be considered a neutral or universally safe management option for contaminated biomass [151]. Where compost derived from metal-enriched plant material is reused in urban landscapes, there is a clear risk of secondary contamination and unintended redistribution of PTEs [152]. For this reason, composting should only be considered where metal concentrations are demonstrably low and downstream use is strictly controlled [153]. In contrast, accumulation-oriented phytoremediation can only be justified when the maintenance chain is clearly defined and consistently implemented [154], including targeted harvesting or pruning, physical removal of contaminated biomass, and downstream handling routes that prevent re-entry into the urban green-waste cycle [155]. Depending on local regulatory frameworks and infrastructure, this may involve separate collection, restricted disposal pathways or controlled thermal treatment.

These constraints have direct implications for design and strategy selection [156]. In settings where safe removal and appropriate downstream handling of biomass cannot be guaranteed across maintenance cycles, phytostabilisation and surface containment are typically more realistic and robust objectives than phytoextraction [157]. In such cases, ornamental plantings that maintain durable ground cover, reduce resuspension and limit direct contact with contaminated topsoil are better aligned with routine urban management practices than strategies relying on repeated harvest of contaminated plant material [20,91].

### 3.10. Human Exposure Considerations in Recreational Urban Settings

Ornamental vegetation in cities is intentionally designed to attract people and promote use of public space, which introduces specific exposure considerations when phytoremediation is proposed [158]. In recreational settings, including parks, playgrounds and residential green areas, direct human contact with vegetation and surface soils is frequent and often unavoidable, particularly for children [159,160]. Under these conditions, accumulation-oriented plantings raise additional concerns, because potentially toxic elements translocated into leaves, flowers or other aboveground tissues may increase the likelihood of human contact through touch, incidental ingestion of dust or plant material [161], and maintenance-related handling.

Potential exposure pathways extend beyond direct plant contact [162]. While evidence for substantial transfer of PTEs through pollen to pollinators or humans remains limited and context dependent [163], the presence of metals in reproductive tissues has been reported for some species, suggesting that this pathway cannot be dismissed a priori in high-use environments [164]. Similarly, repeated skin contact with contaminated foliage or resuspended dust originating from leaf surfaces may contribute to low-level but chronic exposure, particularly in settings where vegetation is accessible and intensively used [165,166].

These considerations have direct implications for plant selection and strategy choice. In recreational and high-contact areas, phytostabilisation-oriented designs are generally more appropriate than phytoextraction-focused approaches [167]. Species that retain metals predominantly in the root zone, limit translocation to aboveground tissues, and provide durable surface cover can reduce resuspension and direct contact with contaminated substrates, thereby better aligning with public health protection goals [168]. In contrast, accumulation-focused plantings that translocate metals to leaves or flowers should be restricted to controlled sites with limited access and clearly defined maintenance and biomass-handling pathways [169,170]. Explicitly accounting for human use intensity and contact pathways is therefore essential when translating phytoremediation concepts into urban green infrastructure practice.

### 3.11. Seasonality, Aesthetic Continuity and Functional Performance

Seasonality is a critical but often under-discussed factor in the effectiveness of ornamental phytoremediation in urban environments [171]. Annual plantings can provide high biomass and measurable uptake during the growing season [172], but they leave soils exposed after senescence and removal, typically during autumn and winter [173]. In temperate cities, this period coincides with increased wind exposure, surface drying and, in some cases, intensified resuspension of contaminated particles, which can counteract exposure-mitigation goals [174].

Perennial and woody ornamentals offer structural continuity across seasons and can reduce these risks by maintaining permanent or semi-permanent ground cover and root systems [146]. Dense perennial covers, grasses and low shrubs limit bare soil exposure, stabilise the upper soil layer and reduce the mobilisation of fine particles during dormant periods [175]. From both a functional and aesthetic perspective, such continuity aligns better with the expectations placed on urban green infrastructure, where visual acceptability, year-round performance and low maintenance demand are central constraints [176].

These considerations suggest that species selection should not be based solely on peak-season accumulation metrics. Instead, seasonal dynamics and off-season performance should be treated as integral components of phytoremediation design [117]. In many public space settings, perennial systems may provide more robust exposure reduction over annual management cycles than annual plantings, particularly where winter soil exposure would otherwise increase resuspension or human contact with contaminated substrates [117].

### 3.12. Invasion Risk Screening for Phytoremediation-Oriented Ornamentals

The invasion potential of ornamental species represents a critical ecological constraint for urban phytoremediation and must be considered alongside remediation performance [92,177]. Many fast-growing or stress-tolerant taxa that show favourable uptake or stabilisation traits under contaminated conditions are non-native and may possess high invasive capacity, particularly in disturbed urban environments [178]. In such contexts, intentional planting can facilitate spread beyond target sites, potentially generating ecological impacts that outweigh the benefits of local contaminant management.

Urban phytoremediation therefore requires an explicit invasion risk screening step prior to species selection [179]. From a precautionary perspective, recommendations should prioritise native species or taxa with a documented history of safe use and no evidence of invasive behaviour within the relevant climatic region [180]. This is particularly important in cities, where propagule pressure is high, habitat disturbance is frequent, and dispersal pathways are numerous [181]. The use of exotic ornamentals with uncertain invasion status should be avoided, even if short-term phytoremediation indicators appear favourable under field conditions [103].

In practice, invasion risk should be treated as a hard constraint rather than a secondary consideration. Species selection for phytoremediation-oriented plantings should therefore be limited to taxa that satisfy both functional criteria (e.g., stabilisation capacity, surface cover, tolerance to mixed PTE exposure) and ecological safety criteria [182]. Integrating invasion risk screening into the design process helps ensure that phytoremediation interventions do not create long-term ecological liabilities while addressing urban contamination [183].

### 3.13. Economic Feasibility and Management Trade-Offs

Economic feasibility is a decisive factor for planners and municipalities considering phytoremediation as part of urban green infrastructure. Unlike engineering-based interventions, phytoremediation operates on multi-year time horizons and relies on repeated maintenance actions, including planting, irrigation, pruning, mowing and the handling of contaminated biomass [184]. These recurring costs need to be weighed against one-off interventions such as soil excavation and replacement, which involve high upfront expenditure for material removal, transport and disposal but provide immediate risk reduction [185].

In practice, neither approach is universally cheaper. Soil replacement may be economically justified for small, highly contaminated hotspots or for sites where rapid risk elimination is required [186]. In contrast, phytoremediation-oriented designs can be cost-competitive where contamination levels are moderate, areas are extensive, and vegetation maintenance is already part of routine management [187]. In such cases, costs associated with planting and long-term care may be partially offset by co-benefits, including microclimate regulation, aesthetic value and recreational functions, which are not delivered by soil replacement [89,188].

Importantly, accumulation-based strategies tend to incur higher long-term costs than stabilisation-oriented designs, because they require repeated biomass removal and controlled disposal to prevent secondary contamination [189]. From an economic perspective, phytostabilisation and surface containment often represent the most robust compromise between cost, risk reduction and management feasibility in urban settings [190]. Explicit consideration of time horizon, contamination intensity and maintenance capacity is therefore essential when comparing phytoremediation with soil replacement as alternative management options [191,192].

## 4. Materials and Methods

### 4.1. Literature Search and Study Selection

We conducted a systematic literature search following PRISMA guidelines (Preferred Reporting Items for Systematic Reviews and Meta-Analyses). The study selection process is summarised in a PRISMA flow diagram (Figure S1) [193], which shows the number of records identified, screened, assessed at full-text level and retained, together with the main reasons for exclusion. A formal review protocol was not registered; however, eligibility criteria, screening steps and extraction variables were pre-defined prior to full-text assessment and applied consistently throughout the workflow. We targeted peer-reviewed, English-language urban field studies published between 2000 and 2025 that reported phytoremediation-relevant outcomes and study-design features for ornamental and horticultural plant species. The search aimed to capture field-based reporting and design patterns relevant to urban ornamental-plant deployment rather than to synthesise harmonised concentration-based remediation effectiveness or to provide a purely bibliometric overview.

Searches were performed on Google Scholar (via Publish or Perish) and the Web of Science Core Collection, with results screened to retain English language records only. Searches were limited to title/abstract/keyword fields where available; Google Scholar searches were executed via Publish or Perish using the same query string. The literature search was conducted between 1 June 2025 and 30 September 2025. Because Google Scholar and Web of Science differ in indexing rules and metadata structure, we focus subsequent analyses on study-level reporting and design patterns and avoid interpreting database coverage as an unbiased representation of worldwide urban field practice. The following Boolean query was used:

(“ornamental plant” OR “urban vegetation” OR “urban green space” OR “roadside plant” OR “horticultural species”) AND (“phytoremediation” OR “heavy metal*” OR “lead” OR “cadmium” OR “zinc” OR “copper” OR “nickel”) AND (“city” OR “urban”)

The search yielded 517 potentially relevant records. Duplicates were identified and removed by matching DOI (when available) and exact/near-exact title strings; ambiguous cases were resolved using first-author and publication-year checks. Records were treated as duplicates when they referred to the same journal article (identical DOI or identical title with matching first author and year). Titles and abstracts were screened first, followed by full-text assessment using predefined inclusion and exclusion criteria. We restricted the synthesis to peer-reviewed journal articles. Restricting the search to English language, peer-reviewed journal articles indexed on Google Scholar and Web of Science may introduce language and database bias in a geographically uneven field, where relevant evidence is also published in local languages and non-indexed outlets. Accordingly, we interpret the synthesis as an evidence map of the English-indexed peer-reviewed literature, and we treat geographic coverage and PTE co-reporting profiles as conditional on database indexing and language rather than as global estimates. Conference abstracts, theses and technical reports were excluded a priori to avoid non-comparable reporting standards and incomplete methodological detail.

Studies were included if they:

- were conducted in urban environments (e.g., roadside strips, parks, wastewater-affected sites, industrial or post-industrial green areas);
- examined ornamental or horticultural plant species commonly used in urban landscaping;
- addressed phytoremediation-relevant processes such as accumulation, translocation, tolerance, or stabilisation;
- evaluated at least one potentially toxic element (PTE; e.g., Pb, Cd, Zn, Cu, Ni);
- reported quantitative data (e.g., tissue or soil concentrations, bioconcentration factors, translocation factors).

Studies were excluded if they:

- were controlled laboratory or hydroponic experiments without a real urban context;
- focused on non-urban or non-ornamental taxa (e.g., agricultural crops, forest trees outside urban settings, invasive weeds);
- were reviews, theoretical papers, or lacked primary quantitative data.

For each included study, we extracted: title; authors; year of publication; plant species and growth form; target elements; environmental context (e.g., roadside, park, wastewater-affected, industrial); primary mechanism focus (accumulation, translocation, stabilisation); DOI; and a brief rationale for inclusion.

Two reviewers independently screened titles and abstracts and independently assessed full texts for inclusion; the same two reviewers then performed independent data extraction and coding, and disagreements were resolved by discussion and re-checking the original article until consensus. Disagreements were resolved prior to analysis. To minimise classification drift, coding rules were documented a priori, and inter-rater agreement was tracked (percent agreement); disagreements were adjudicated by returning to the primary source and agreeing on a final code.

The presence or absence of each target element and each reported mechanism category was coded in binary format (yes/no). Outcome variables describing reporting breadth—such as metal richness (PTE richness) and the number of target elements per study—were coded numerically. After screening and coding, 167 studies met the inclusion criteria and formed the basis of the quantitative synthesis of reporting patterns across studies.

For the purposes of this review, we defined “ornamental/horticultural” species as plants intentionally used for decorative, amenity or landscaping purposes in public or private urban green spaces, including ornamental shrubs and trees, flowering annuals and perennials, turf and groundcovers. Fruit trees and edible species were included only when they were clearly described as part of ornamental plantings rather than as agricultural crops. Sites were classified as “urban” when located within the administrative boundaries of towns or cities, including peri-urban districts that form part of the contiguous built-up area; purely rural or agricultural settings were excluded even when similar plant species were cultivated.

Each eligible study contributed one observational unit to the quantitative analyses, defined at the study level. Where a paper reported multiple species and/or multiple sampling locations, we assigned plant functional type and environment type using a pre-defined dominant-category rule: we prioritised the primary target species (as stated by the authors) and the primary environment/site type (i.e., the main setting framing the study objectives); when this was ambiguous, we selected the category with the largest reported sample size. The PTE profile was then summarised at the study level. This design limits artificial inflation of the effective sample size due to non-independent within-study contrasts (multiple species, sites, or tissues) and maintains a consistent unit of analysis for the regression and contingency models. To evaluate potential bias from within-study heterogeneity, we report how often multi-species and multi-site designs occurred and, in sensitivity checks, verified that the main association patterns were not driven by these studies; species- and site-specific contrasts are discussed qualitatively in the Discussion. We therefore interpret the quantitative results as study-level reporting patterns and contextual associations, acknowledging that they do not resolve within-study variability in uptake or tissue partitioning.

### 4.2. Statistical Analyses

Statistical analyses were performed in IBM SPSS Statistics v26 (IBM Corp., Armonk, NY, USA) and validated in R v4.3.1 (R Foundation for Statistical Computing, Vienna, Austria). We first summarised evidence patterns across plant functional types, environmental categories and PTE profiles using descriptive statistics. Because several variables deviated from normality (Shapiro–Wilk test, *p* < 0.05), we applied non-parametric tests to compare metal richness across environments (Kruskal–Wallis H test with post hoc pairwise Mann–Whitney U tests and Bonferroni correction).

To examine recurring element combinations and broad evidence structures across urban contexts, we applied correlation summaries and multivariate ordination focused on co-reporting. Because PTE information was frequently reported as presence/absence rather than harmonised concentration endpoints, we constructed a study-by-element binary matrix indicating whether each element was analysed in each study; elements not analysed in a given study were coded as 0. The element set used in these analyses is provided explicitly (Pb, Cd, Zn, Cu, Ni, Cr, Fe, Mn, Hg, As, B). Pairwise associations among elements were summarised using Pearson correlations computed on the binary matrix; for binary variables this statistic is equivalent to the Phi coefficient and is therefore interpretable as a measure of co-reporting association across studies. These correlations were interpreted descriptively as evidence-structure signals shaped by reporting practices and study design, not as proof of shared sources, mechanistic coupling, or uptake kinetics.

Principal component analysis (PCA) was then applied to the element-by-study presence/absence information to reduce dimensionality and identify dominant co-reporting profiles of PTE assessment. Components were retained using the Kaiser criterion (eigenvalues > 1) and rotated with Varimax to improve interpretability of element loadings. Given the lack of harmonised concentration data and endpoints, PCA outputs were used to summarise co-reporting structure in the evidence base rather than to infer mechanistic dependence among elements.

The resulting principal component scores were treated as continuous proxies for PTE mixtures and served as inputs to a linear discriminant analysis assessing whether environment categories occupy distinct regions of this multivariate space. We recognise that this application assumes approximate linearity and abstracts from concentration gradients, and we therefore interpret PCA and discriminant outputs as exploratory descriptors of co-reporting patterns rather than as predictive models of uptake.

Finally, we used linear and multiple regression models to assess how publication year, environment type, plant type, and mechanism focus were associated with metal richness (PTE richness) as an index of reporting breadth. Associations among categorical variables (e.g., plant type × mechanism focus; environment × mechanism focus) were examined using cross-tabulations and Pearson’s chi-square tests, with effect sizes quantified by Cramér’s V (and Phi where applicable). A significance threshold of *p* < 0.05 was applied; where families of related tests were run, *p*-values were adjusted for multiple testing. In all regression models, the study was the unit of analysis: each of the 167 studies contributed a single data point characterised by its PTE richness and study-level descriptors. This structure minimises pseudo-replication and aligns with our focus on how study context and design shape the breadth of PTE assessment and reporting in urban ornamental phytoremediation research, rather than quantifying contamination magnitude or remediation performance. We did not synthesise concentration-derived performance metrics (e.g., tissue concentrations, BCF, TF) because reporting was not sufficiently standardised across studies; therefore, our quantitative synthesis is limited to study-level reporting patterns and contextual correlates.

## 5. Conclusions

This review summarises field-based evidence on ornamental and horticultural species used in urban contexts, focusing on how phytoremediation-related functions are framed and reported under PTE exposure. Across 167 studies, reported outcomes and the breadth of assessed elements differed mainly by site context and multi-element settings, while consistent field-based evidence for ranking individual species by performance remained limited. Ornamental plants may therefore be considered within urban risk-management strategies as components of site-matched, functionally designed plantings, while recognising that this synthesis addresses reporting patterns rather than quantified exposure reduction or long-term stabilisation. PTE richness showed its strongest association with environment type, capturing heterogeneity in urban contamination contexts as well as differences in which elements are typically assessed and reported across settings. Wastewater-affected and industrially influenced settings tended to show broader multi-element profiles than parks or roadside belts. This supports environment-based organisation of evidence that prioritises local contamination profiles and exposure pathways in study design and interpretation, while avoiding generic species rankings based on heterogeneous field reports.

The literature also shows a clear imbalance in mechanism focus. Accumulation dominates, in line with a strong preference for aboveground sampling, while stabilisation and translocation are less often evaluated under field conditions. Current knowledge may thus understate rhizosphere-level processes that are central to long-term exposure reduction, even though field-based evidence for quantified exposure reduction or stabilisation effectiveness remains limited. More consistent root-zone profiling and harmonised reporting of key soil properties would improve cross-study comparability and provide a firmer basis for context-specific applied recommendations.

Given that most field studies emphasise aboveground accumulation endpoints, a precautionary planning implication is to favour stabilisation- and containment-oriented plantings in high-traffic, intensively used urban green spaces. Accumulation- or hyperaccumulator-oriented plantings may be considered only in controlled, low-access locations (e.g., fenced traffic islands). They should be used only with clearly defined and locally feasible biomass-removal and safe-handling protocols. Without managed removal, uptake into biomass does not imply risk reduction.

In summary, our findings argue for functionally diverse, site-specific planting systems that combine woody ornamentals, herbaceous species and perennial ground covers, as a plausible direction for designs intended to accommodate heterogeneous urban PTE contexts. Such plantings are more likely to match heterogeneous urban PTE profiles and to fit into urban green infrastructure with realistic management demands. Future work that couples standardised soil property reporting with balanced root–shoot sampling will be important for moving from heterogeneous field reports toward a more comparable evidence base, which is a prerequisite for any robust risk-management claims. These conclusions should be read in light of the methodological choices made: PTE richness is a coarse indicator that does not reflect concentration gradients, and the presence/absence coding in our multivariate analyses ignores dose–response relations. These practice-oriented considerations are intended as decision support for green infrastructure planning under heterogeneous urban PTE conditions, rather than as prescriptive remediation protocols. Our typology is therefore best seen as an exploratory tool for organising field evidence and describing reporting patterns, rather than as a predictive model of site behaviour or remediation effectiveness.

## Figures and Tables

**Table 1:** Phytoremediation-related mechanisms of ornamental plants, indicating plant parts and urban exposure implications based on metal retention in roots versus accumulation in leaves or flowers.

Mechanism Type	Functional Trait/Mechanism	Description	Ecological/Remediation Outcome	Plant Part	Urban Exposure Implication	Reference
Biochemical	Antioxidant defence systems	Increased activity of SOD, POD, CAT	Mitigates oxidative stress, maintains growth under heavy metal exposure	Whole plant tissues	Context dependent	Liu et al. [49], Liu et al., [93], Singh et al. [94]
	Metal sequestration and detoxification	Compartmentalization in vacuoles or binding to cell walls	Reduced cytotoxicity, long-term tolerance	Root tissues and shoots	Context dependent	Bhaduri & Fulekar, [95], Li et al. [56]
Morphological/structural	Leaf microstructures, dense canopy, trichomes	Interception of dust and particulate-bound metals (phylloremediation)	Reduction in airborne particulate metals, surface deposition control	Leaves, leaf surface	Higher contact risk	Santunione et al. [96], Wei et al. [97]
Physiological	Metal uptake and translocation	Root-based absorption and movement to shoots via ion transporters and chelators (e.g., organic acids, phytochelatins)	Extraction of metals into harvestable biomass	Root to shoot pathway	Higher contact risk	Biswal et al. [98]; Singh et al. [94]
	Soil–root–microbe interactions	Associations with mycorrhizal fungi and plant growth-promoting rhizobacteria	Enhanced metal solubility and uptake	Rhizosphere and roots	Context dependent	Ștefan et al. [99], Rasouli et al. [100], Gul et al. [101]

**Table 2:** Summary statistics for PTE richness and regression models describing the number of PTEs assessed per study.

Component	Metric/Category	Value/Description
PTE richness	Mean number of PTEs per study	3.21
	Standard deviation (SD)	1.37
	Range	1–7 PTEs/study
Morphological/structural	Core elements	Pb, Cd, Zn
	Frequency of core elements	Present in 67% of studies
	Less frequently assessed elements	Ni, Cr, B (each reported in <10% of studies)
Environment type distribution	Roadside green belts	34.3% of studies
	Wastewater-affected sites	28.4% of studies
	Urban parks	22.5% of studies
	Industrial/post-industrial and other urban sites	14.8% of studies
Mechanism focus (dominant per study)	Accumulation	56.8% of studies
	Translocation (root–shoot transfer, TF-based)	25.3% of studies
	Stabilisation (rhizosphere/soil immobilisation)	17.9% of studies
Sampling emphasis	Aboveground tissues (shoots, leaves)	Assessed in 73.4% of studies
	Root profiles/root-zone	Assessed in 28.9% of studies
PCA of PTE presence/absence	Variance explained by first three principal components	64.5%
	PC1 profile	High loadings of Pb, Zn, B (“traffic/construction” mixture)
	PC2 profile	Cu and Ni (“industrial abrasion”)
	PC3 profile	Cd and Cr (“wastewater/industrial discharge”)
Regression models for PTE richness	Model 1: publication year only	F(1,167) = 0.009, *p* = 0.924 (not significant)
	Model 2: year + plant type + environment type + mechanism focus	F(4,164) = 4.651, *p* = 0.001; Adjusted R^2^ = 0.08

## Data Availability

Data is available on request.

## Supplementary Materials

The following supporting information can be downloaded at: https://www.mdpi.com/article/10.3390/plants15040662/s1, Figure S1: PRISMA diagram.

## Abbreviations

The following abbreviations are used in this manuscript:

PTE	potentially toxic element
BCF	bioconcentration factor
TF	translocation factor
