# Supplementary figures and images for: Ornamental Phytoremediation in Cities: Context-Dependent Roles in Managing Potentially Toxic Elements
Source: Plants (Basel). 2026 Feb 22;15(4):662. doi: 10.3390/plants15040662 (PMC12944677; doi:10.3390/plants15040662)

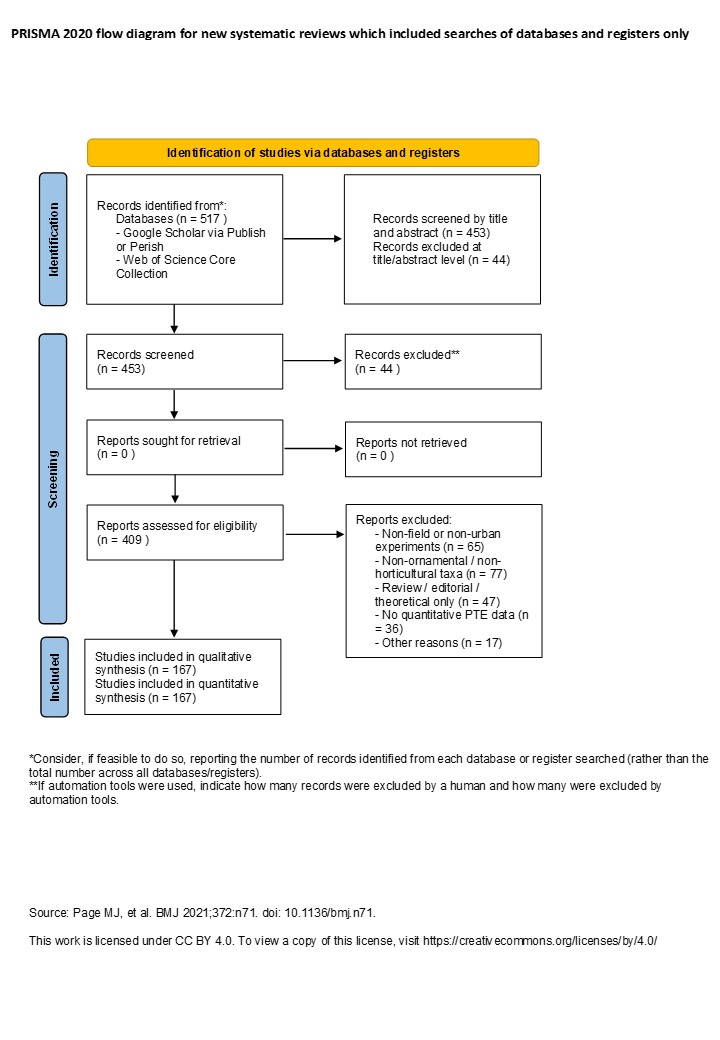

Supplement: Supplementary file 1 [file plants-15-00662-s001.zip › plants-4133727-supplementary.jpg]
